# How Does the Dietary Intake of Female Field-Based Team Sport Athletes Compare to Dietary Recommendations for Health and Performance? A Systematic Literature Review

**DOI:** 10.3390/nu13041235

**Published:** 2021-04-09

**Authors:** Michèle Renard, David T. Kelly, Niamh Ní Chéilleachair, Ciarán Ó Catháin

**Affiliations:** 1 Department of Sport and Health Sciences, Athlone Institute of Technology, N37 HD68 Athlone, Ireland; davidkelly@ait.ie (D.T.K.); nnicheilleachair@ait.ie (N.N.C.); ciaranocathain@ait.ie (C.Ó.C.); 2 SHE Research Group, Athlone Institute of Technology, N37 HD68 Athlone, Ireland

**Keywords:** sports nutrition, nutritional recommendations, carbohydrate intake, energy, LEA, female health

## Abstract

Field-based team sports present large energetic demands given their intermittent high-intensity nature. Current evidence suggests that the dietary intake of female athletes may be insufficient to meet such demands, resulting in negative consequences for athletic performance and health. The primary aim of this review was to therefore assess the adequacy of dietary intake of female field-based team sport athletes when compared to dietary recommendations. A systematic search of databases, including PubMed, Web of Science, SPORTDiscus, and OpenGrey, was performed from the earliest record available until July 2020, obtaining an initial total of 2588 articles. To be included within the final review, articles were required to provide a quantitative assessment of baseline dietary intake specific to the target population. A total of 20 studies (*n* = 462) met the full eligibility criteria. A majority reported that the dietary intake of female field-based team sport athletes was insufficient in overall energy (2064 ± 309 kcal·day^−1^), carbohydrate (4.3 ± 1.2 g·kg·day^−1^), and iron intake (13.6 ± 6.2 mg·day^−1^) when compared to recommendations. Future research is required to establish why female team sport athletes consistently demonstrate deficient dietary practices, and to explore the potential negative consequences of this.

## 1. Introduction

Field-based team sports are characterized by intermittent high-intensity activity, followed by periods of low-to-moderate active recovery or passive rest [1,2,3,4,5]. The exact characteristics of each sport vary from those that are more strength and power-focused (American football and rugby) to those with greater emphasis on endurance (soccer, field hockey, lacrosse, Australian rules, and Gaelic football) [4,5]. The gameplay of all field-based team sports places demands on both aerobic and anaerobic systems of energy production, leading to the depletion of muscle glycogen stores [6]. Therefore, nutritional strategies to ensure glycogen stores are sufficient to meet the energy costs of training and competition are highly recommended to delay the onset of fatigue, optimize performance during gameplay, and support physiological adaptation and recovery [6,7,8,9].

Despite the broad publication of dietary recommendations for athletes [8,9,10], female field-based team sport athletes have repeatedly been shown to consume diets that are insufficient in energy intake during competition [11,12] and training periods [13,14], largely explained by a failure to meet recommendations for carbohydrate intake [11,12,13,14,15,16]. Such inadequate dietary intake in female athletes has not only been associated with decreased athletic performance, but also a multitude of acute and chronic health issues ranging from menstrual dysfunction [17,18,19] and suboptimal bone health [17,18,19] to increased risk of injury and illness [19]. The concept of low energy availability (LEA), whereby an individual has inadequate energy intake relative to their energy expenditure (EE) [20,21], has recently been identified to occur frequently, not just in weight-sensitive and endurance-based athletes, but also those that compete in team sports [22,23].

It has been suggested that measuring adherence to macronutrient recommendations [9,24] may support the monitoring of energy status and identification of LEA risk among female athletes [25]. Given the prevalence of micronutrient deficiencies with LEA [22], and the existing risk of deficiency among female athletes [26,27], it may be pertinent for micronutrient intake to also be explored in this context. From a total of 64 studies investigating the dietary intake of team sport athletes captured in previous narrative [5] and systematic reviews [28], only 29.7% (*n* = 19) included female athletes and 14.1% (*n* = 9) took part in field-based team sports. An increasing number of dietary intake observations in female field-based team sport athletes now exist [12,14,15,16,29,30,31,32], which have not yet been subject to review. Given the distinct physiological requirements of field-based team sports [1,2,3,4,5], and the increased requirement to address issues of dietary inadequacy and LEA within female athletes [17,18,19,21,27], a systematic review focusing specifically on this population is warranted.

Therefore, the primary aim of this review was to assess the adequacy of dietary intake in female field-based team sport athletes when compared to dietary recommendations for maintenance of general health [33,34] and optimal sporting performance [8,9,10].

## 2. Materials and Methods

### 2.1. Protocol Registration

All methods of the review were conducted in accordance with the preferred reporting items for systematic reviews and meta-analyses (PRISMA) guidelines [35]. The review protocol was registered with the international prospective register of systematic reviews (PROSPERO) before the formal screening of search results against eligibility criteria (registration number: CRD42020197673) [36]. The PICOS (participants, intervention, comparison, outcome, study design) criteria applied to the review are outlined in Table 1.

### 2.2. Search Strategy

A comprehensive search strategy designed to identify both commercially published articles and sources of grey literature was developed with assistance from an experienced health science librarian. A systematic search of databases, including PubMed (MEDLINE), Web of Science (core collection including conference proceedings), SPORTDiscus, and OpenGrey (grey literature database), was performed by one author (MR) from the earliest record available until July 2020. Examples of search terms used included: “dietary intake”, “dietary assessment”, “field-based sport”, and “team-sport athlete”. The full list of search terms used is detailed in Table 2.

### 2.3. Eligibility Criteria

All original research, including observational, cross-sectional, and randomized control trials, which provided a measurement of total energy (MJ/day, kcal/day) and macronutrient intake (carbohydrate, fat, and protein) were considered for inclusion. Studies that did not report a baseline assessment of dietary intake and studies that only provided a qualitative assessment of dietary intake were excluded. Only articles published in English were included; unpublished theses, conference posters, and abstracts were considered if all other inclusion criteria were met. Each study included was required to have reported data specifically regarding female athletes that competed in field-based invasion team sports. Field-based invasion team sports were defined as games played on a field/pitch by two opposing teams, with the primary objective of invading the oppositions territory to score points [37] (e.g., field hockey, soccer, lacrosse, rugby). Data reported in an aggregated format, whereby the specific target population was indistinguishable from others, were excluded. All age divisions (i.e., youth and adult) and competitive levels (i.e., amateur and professional) that met the above criteria were accepted. A full list of the inclusion and exclusion criteria that were applied is detailed within Table 3.

### 2.4. Study Selection Process

**Figure 1:**
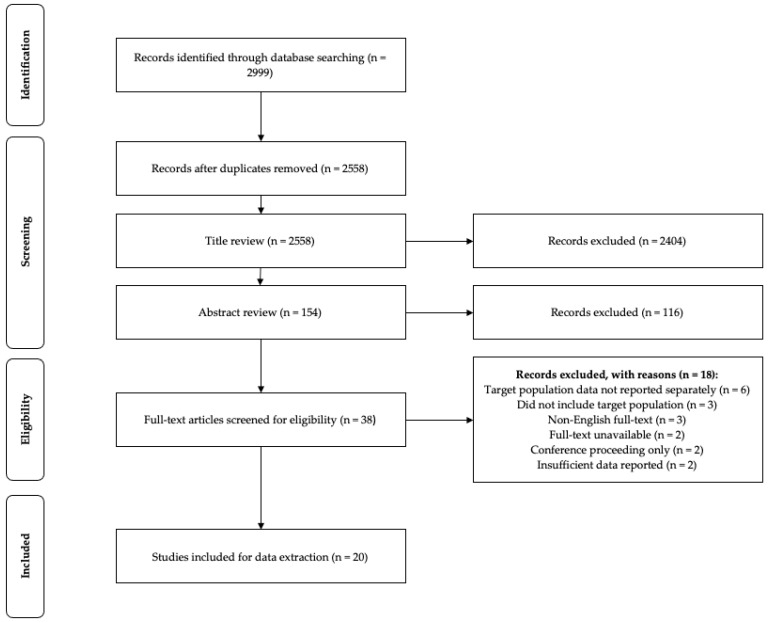
Study selection process.

Studies were initially screened based on title and abstract content by two authors (MR and COC). Duplicates and studies clearly unrelated to the review topic were removed at this stage. All articles that progressed from the title and abstract review were retrieved for full-text review. Each full text was screened against the inclusion and exclusion criteria outlined in Table 3 by two authors (MR and COC). Any disagreement surrounding article eligibility between the two independent reviewers at this stage was reviewed by additional authors (DK and NNC). Finally, the reference list of all articles that passed full-text review and were deemed eligible for data extraction were checked by two authors (MR and COC) and manual searches were conducted using Google Scholar to ensure no relevant articles were overlooked. The selection and exclusion of studies for each stage are reported in Figure 1.

### 2.5. Data Extraction

Data were extracted from the eligible studies by two authors (MR and COC). This included: study background (country, sport, time of season, participant demographic information (athletic level, age (years)), anthropometric measures (height (cm), total mass (kg), BMI (kg·m^2^), lean mass (kg), body fat (%)), method and duration of dietary assessment used (i.e., 3-day estimated food record), method of energy expenditure assessment if measured (e.g., accelerometry), total energy expenditure (kcal·day^−1^), total energy intake (kcal·day^−1^), carbohydrate, protein and fat intake (g, g·kg^−1^·day^−1^, % total daily energy intake (TDEI), and where provided, iron, calcium (mg·day^−1^), and vitamin D (µg·day^−1^) intake. If energy was only reported in KJ/MJ, it was converted into kcal·day^−1^ [15,38]. Similarly, if carbohydrate, protein, and fat intake were not reported in the required units, these were converted to g, g·kg·day^−1^, or % TDEI [11,12,13,14,30,31,32,39,40,41,42,43] to facilitate comparison across common units and against recommendations [8,9,10].

### 2.6. Quality Assessment

The quality of each full-text article that was deemed eligible for data extraction was assessed independently by two authors (MR and COC). Any disagreement surrounding the quality rating allocated to each study at this stage was reviewed by additional authors (DK and NNC). Studies were reviewed using the Academy of Nutrition and Dietetics quality criteria checklist [44], which permits the assessment of relevance and validity, with the allocation of either a positive, neutral, or negative quality ranking. Questions that referred to study group comparisons, methods for handling withdrawals, and the use of blinding and intervention descriptions were not applied as all studies included were either observational or cross-sectional in design. Therefore, the key criteria to achieve a positive rating were a thorough outline of study procedures, evidence of an inclusion/exclusion criteria applied, use of methods to reduce selection bias, and reporting of potential confounding factors on the outcome being measured (dietary intake).

## 3. Results

### 3.1. Study Selection

The search strategy obtained a total of 2999 articles for review, 2558 after the removal of duplicates, 154 after title screening, and 38 after abstract screening. Of the 38 articles designated for full-text review, 20 [11,12,13,14,15,16,25,29,30,31,32,38,39,40,41,42,43,45,46,47] met the criteria to proceed to data extraction and were included in the systematic review. Reasons for article exclusions are highlighted in Figure 1. No additional studies were identified through hand searching of reference lists or manual searches using Google Scholar.

### 3.2. Quality Assessment

One study received a positive quality rating [32] whilst all remaining studies received a neutral rating [11,12,13,14,15,16,25,29,30,31,38,39,40,41,42,43,45,46,47]. No studies received a negative rating, therefore quality assessment did not eliminate any articles obtained. Studies that only received neutral ratings were mostly affected by failure to specify an inclusion/exclusion criterion for the study population, and by extension, failure to provide sufficient information to prove the study participants were a representative sample of the target population. In addition, few studies accounted for other factors that could affect the outcome (dietary intake), for example, risk/prevalence of disordered eating within the population and the potential impact this could have on the values reported.

### 3.3. Study Characteristics

Studies were observational in design and either cross-sectional (*n* = 14) [11,12,13,14,15,16,29,30,38,41,42,43,46,47] or longitudinal (*n* = 5) [25,31,32,39,45], with the addition of a randomized controlled trial (*n* = 1) [40] where baseline data prior to the intervention was available. The majority of female field-based team sport athletes included in this review were from the United States of America (*n* = 105) [12,13,25,30,31,39,45], Germany (*n* = 56) [47], Australia (*n* = 52) [15,16], and the United Kingdom (*n* = 45) [11,29,43], with the remaining studies including athletes from Poland (*n* = 41) [14], Canada (*n* = 33) [46], Spain (*n* = 28) [40], Brazil (*n* = 21) [41], Japan (*n* = 17) [32], the Netherlands (*n* = 16) [38], and Iran (*n* = 8) [42]. The majority of female field-based team sport athletes included in this review were soccer players (*n* = 288) [11,13,14,25,29,30,38,40,41,42,45,46,47], followed by lacrosse (*n* = 57) [12,31,32], Australian rules football (*n* = 52) [15,16], rugby sevens (*n* = 29) [38], field hockey (*n* = 20) [38,39], and touch rugby players (*n* = 16) [43]. Of these athletes, many competed at a national/professional level *(n* = 351) [11,13,14,15,16,29,38,40,41,42,43,46,47], whilst the remaining competed at a varsity/collegiate level (*n* = 111) [12,25,30,31,32,39,45]. Studies included both adult (*n* = 338, age = 21.1 ± 2.4 years) [11,12,13,14,15,16,25,29,30,31,32,38,39,40,41,43,45] and youth athletes (*n* = 124, age = 15.6 ± 2.0 years) [38,42,46,47]. The anthropometric averages for all athletes included were height (165.9 ± 3.2 cm), total mass (62.3 ± 5.0 kg), BMI (22.6 ± 1.3 kg·m^2^), lean mass (46.9 ± 1.7 kg), and body fat (19.9 ± 5.6%) [11,12,13,14,15,16,25,29,30,31,32,38,39,40,41,42,43,45,46,47].

Studies mostly measured dietary intake “in-season” (*n* = 12) [11,12,14,29,30,31,38,39,40,42,46,47] and “pre-season” (*n* = 7) [15,16,25,31,32,41,45]. Some also measured dietary intake “post/off-season” (*n* = 5) [25,31,32,39,45], across a competitive 4-day tournament (*n* = 1) [43], and during an unspecified training period (*n* = 1) [48]. Dietary intake was measured most frequently using “estimated” food records over 3 days (*n* = 5) [13,14,16,25,45], 4 days (*n* = 5) [12,30,31,43,46], and 7 days (*n* = 4) [11,32,39,47]. Only two studies specifically stated that they used “weighed” food records over 5 days (*n* = 1) [29] and 8 days (*n* = 1) [40]. The remaining studies relied on multiple 24-h recalls, which all captured 3 days’ worth of dietary intake data (*n* = 4) [15,38,41,42]. All studies included information on total energy and macronutrient intake (*n* = 20) [11,12,13,14,15,16,25,29,30,31,32,38,39,40,41,42,43,45,46,47] as per the inclusion criteria. Half of these also provided data on micronutrient intake (*n* = 10) [11,13,14,15,16,32,42,45,46,47], with most reporting iron (mg·day^−1^), calcium (mg·day^−1^), and vitamin D (µg·day^−1^) (*n* = 8) [11,13,14,32,42,45,46,47], and two just reporting iron and calcium (*n* = 2) [15,16]. A number of studies included also provided values for energy expenditure (*n* = 11) [11,12,14,30,31,32,38,41,43,46,47]. Some provided estimates based on predictions of basal/resting metabolic rate in combination with estimates of exercise energy expenditure from activity logs and allocation of the metabolic equivalent of tasks (METs) (*n* = 5) [11,38,41,46,47], others utilized accelerometer technology (*n* = 5) [12,14,30,31,32], and one used predictive equations, accelerometry, global positioning system technology, and metabolic power equations combined (*n* = 1) [43]. Study characteristics are reported in Table 4.

### 3.4. Dietary Intake of Field-Based Team Sport Athletes

#### 3.4.1. Total Energy Intake

The average total daily energy intake reported from all studies, including those with longitudinal designs (multiple values), was 2064 ± 309 kcal·day^−1^ (33.4 ± 6.6 kcal·kg·day^−1^), with a range between 1426 and 3122 kcal·day^−1^ (22.4–58.6 kcal·kg·day^−1^) [11,12,13,14,15,16,25,29,30,31,32,38,39,40,41,42,43,45,46,47]. A majority of studies (*n* = 12) [11,12,13,14,25,29,30,31,39,41,46,47] deemed daily energy intake as inadequate to meet demands, few highlighted total energy intakes as sufficient (*n* = 3) [42,43,45], and the remaining studies (*n* = 5) [15,16,32,38,40] did not comment on the adequacy of energy intake directly. A majority of studies (*n* = 7) [12,14,30,31,41,43,47] evaluated the adequacy of energy intake based on comparisons with measures/estimates of energy expenditure, whilst others compared against recommendations of 47–60 kcal·day^−1^ for female soccer players [49] (*n* = 3) [11,13,46], dietary reference values of 37 kcal·day^−1^ [50,51] (*n* = 2) [39,45], cut-off values for reduced energy availability of 30–45 kcal·day^−1^ [52] (*n* =1) [29], and low energy availability of <30 kcal·day^−1^ [18,53] (*n* = 2) [25,29]. One study deemed the energy intake of athletes as sufficient; however, it did not highlight how it was evaluated (*n* = 1) [42]. Only two studies provided comparisons of energy intake across training and recovery days (*n* = 2) [16,29], one of which also included comparisons with a competition day (*n* = 1) [29]. Both sets of comparisons [16,29] revealed no significant differences in energy intake (kcal·day^−1^) between days.

#### 3.4.2. Total Energy Expenditure

The average total daily energy expenditure from studies that reported a measured/estimated value (*n* = 11) [11,12,14,30,31,32,38,41,43,46,47], including those with longitudinal designs (multiple values), was 2543 ± 301 kcal·day^−1^, with a range between 1744 and 3131 kcal·day^−1^. No studies reported comparisons of total daily energy expenditure (kcal·day^−1^) between training, competition, and recovery days.

#### 3.4.3. Macronutrient Intake

The average carbohydrate intake from all studies [11,12,13,14,15,16,25,29,30,31,32,38,39,40,41,42,43,45,46,47] was 4.3 ± 1.2 g·kg·day^−1^, with a range between 2.7 and 8.3 g·kg·day^−1^. A majority of studies (*n* = 13) [11,12,13,14,15,16,29,30,31,38,39,40,43] reported an average daily carbohydrate intake below recommendations for a moderate exercise program (1h·day^−1^) of 5–7 g·kg·day^−1^ [8,9]. One of these studies [16] reported a daily intake of 2.7 g·kg·day^−1^ during a pre-season training period, which fails to meet recommendations for low-intensity or skill-based activities of 3-5 g·kg·day^−1^ [8,9]. The remaining studies met the recommendation of 5–7 g·kg·day^−1^; however, most (*n* = 4) [32,45,46,47] reported intakes at the lower end of the scale, with a range of 5.0–5.4 g·kg·day^−1^ reported. Only two studies reported values that met the recommendation for a moderate to high-intensity exercise program (1–3 h·day^−1^) of 6–10 g·kg·day^−1^, with values of 7.0 g·kg·day^−1^ reported for NCAA division 1 soccer players (*n* = 19) during a pre-season training block [25] and 8.3 g·kg·day^−1^ reported for youth elite soccer players (*n* = 8) in-season [42].

The average protein intake from all studies [11,12,13,14,15,16,25,29,30,31,32,38,39,40,41,42,43,45,46,47] was 1.4 ± 0.3 g·kg·day^−1^, with a range between 0.9 and 2.0 g·kg·day^−1^. In comparison to protein recommendations of 1.2–2.0 g·kg·day^−1^ [9], a majority of studies (*n* = 16) [11,12,13,14,15,16,29,30,32,38,40,41,42,43,46,47] reported average intakes that met this requirement. The remaining studies (*n* = 4) [25,31,39,45] reported values below the recommendation during off-season periods, with one investigation on field hockey players *(n* = 9) [39] reporting low intakes of 0.95 and 0.9 g·kg·day^−1^ during both in-season and off-season periods, respectively.

The average fat intake from all studies [11,12,13,14,15,16,25,29,30,31,32,38,39,40,41,42,43,45,46,47] was 31.2 ± 3.4% of total daily energy intake (TDEI), with a range between 21% and 37.5%. A majority of studies (*n* = 15) [11,13,14,15,16,25,29,32,38,39,41,42,45,46,47] reported intakes within the recommended range of 20–35% [9]. The remaining studies (*n* = 5) [12,30,31,40,43] reported fat intakes >35% TDEI. Of these, only one reported this for a single time period (in-season) out of the five measured [31].

Only two studies [16,29] provided comparisons of macronutrient intake between training, recovery, and match days. One investigation compared the macronutrient intake of Australian rules football players (*n* = 23) [16] between their main and light training days and a recovery day and identified no significant differences in intake across all macronutrients. The other compared macronutrient intakes of professional soccer players (*n* = 13) [29] between heavy and light training days, a recovery day, and a match day, only identifying a significant difference in relative fat intake, which was higher on the heavy training day in comparison to the light training day.

#### 3.4.4. Micronutrient Intake

The average daily iron intake from studies that reported a value (*n* = 10) [11,13,14,15,16,32,42,45,46,47] was 13.6 ± 6.2 (mg·day^−1^). In comparison to recommendations of 14.8 mg·day^−1^ for females aged 11–50 years [33], only four studies reported intakes that met the requirement [13,42,45,46]. The average daily calcium intake from studies that reported a value (*n* = 10) [11,13,14,15,16,32,42,45,46,47] was 829 ± 226 (mg·day^−1^). In comparison to recommendations of 700 mg·day^−1^ for females aged 19–50 years [33], eight studies reported intakes that met the requirement [11,13,15,16,42,45,46,47]. The average daily vitamin D intake reported (*n* = 8) was 3.1 ± 1.4 (µg·day^−1^) [11,13,14,32,42,45,46,47]. In comparison to recommendations of 10 µg·day^−1^ (400 IU·day^−1^) [34], no studies reported intakes that met this recommendation.

### 3.5. Comparisons between Sub-Groups

#### 3.5.1. Sport

The mean energy (kcal·day^−1^), carbohydrate (g·kg·day^−1^), protein (g·kg·day^−1^), and fat intakes (% TDEI) for each sport are displayed within Table 5.

#### 3.5.2. Competitive Level

Mean energy, carbohydrate, protein, and fat intakes for national/professional-level athletes (*n* = 351) [11,13,14,15,16,29,38,40,41,42,43,46,47] were: 2121 ± 356 kcal·day^−1^, 4.4 g·kg·day^−1^, 1.5 ± 0.3 g·kg·day^−1^, and 30.6 ± 3.9% TDEI; and for varsity/collegiate-level athletes (*n* = 111) [12,25,30,31,32,39,45]: 2011 ± 258, 4.3 ± 1.0 g·kg·day^−1^, 1.3 ± 0.3 g·kg·day^−1^, and 31.8 ± 2.9 % TDEI, respectively.

#### 3.5.3. Adult vs. Youth

Mean energy, carbohydrate, protein, and fat intakes for adult athletes (*n* = 338, age = 21.1 ± 2.4 years) [11,12,13,14,15,16,25,29,30,31,32,38,39,40,41,43,45] were: 2018 ± 257 kcal·day^−1^, 4.1 ± 1.0 g·kg·day^−1^, 1.4 ± 0.3 g·kg·day^−1^, and 31.4 ± 3.7% TDEI; and for youth athletes (*n* = 124, age = 15.6 ± 2.0 years) [38,42,46,47], 2304 ± 470 kcal·day^−1^, 5.4 ± 1.7 g·kg·day^−1^, 1.4 ± 0.1 g·kg·day^−1^, and 30.4 ± 0.8% TDEI, respectively.

#### 3.5.4. Training/Competition Phase

Mean energy, carbohydrate, protein, and fat intakes for “in-season” periods (*n* = 12) [11,12,14,29,30,31,38,39,40,42,46,47] were: 2077 ± 360 kcal·day^−1^, 4.3 ± 1.3 g·kg·day^−1^, 1.4 ± 0.3 g·kg·day^−1^, and 31.3 ± 4.3% TDEI, respectively. For “pre-season” periods (*n* = 7) [15,16,25,31,32,41,45], intakes were: 2120 ± 211 kcal·day^−1^, 4.6 ± 1.6 g·kg·day^−1^, 1.6 ± 0.3 g·kg·day^−1^, and 30.8 ± 2.8% TDEI; and for “post/off-season” periods (*n* = 5) [25,31,32,39,45]: 1919 ± 284 kcal·day^−1^, 4.1 ± 0.8 g·kg·day^−1^, 1.0 ± 0.1 g·kg·day^−1^, and 30.9 ± 0.5% TDEI, respectively. Of the longitudinal studies that measured dietary intake during multiple phases (*n* = 5) [25,31,32,39,45], three [31,32,39] reported a lack of significant differences in energy and macronutrient intake between phases, whilst the remaining two studies [25,45] reported greater energy, carbohydrate, and protein intakes during pre-season in comparison to in-season [25] and post-season [25,45].

## 4. Discussion

This review aimed to assess the adequacy of dietary intake in female field-based team sport athletes when compared to dietary recommendations for maintenance of general health [33,34] and optimal sporting performance [8,9,10].

### 4.1. Energy Balance of Field-Based Team Sport Athletes

A majority of studies (*n* = 12) [11,12,13,14,25,29,30,31,39,41,46,47] concluded that the energy intake of female field-based team sport athletes was insufficient to meet the demands of training and competition. Unfortunately, the magnitude of this insufficiency is difficult to determine due to the heterogeneity of criteria used to evaluate the adequacy of energy intake. For example, the criteria used by one study to declare that the energy intake of soccer players was sufficient (37–41 kcal·kg·day^−1^) [45] would be declared as insufficient in comparison to criteria used by other investigations with soccer players (47–60 kcal·kg·day^−1^) [11,13,46]. Calculations of LEA cut-offs for the interpretation of dietary adequacy were also limited by the lack of fat-free mass (kg) data reported by studies. Evaluation of the energy balance may therefore be sought from studies that also provided a value of energy expenditure. The mean energy expenditure reported of 2543 kcal·day^−1^ [11,12,14,30,31,32,38,41,43,46,47] was greater than the mean energy intake reported of 2064 kcal·day^−1^ [11,12,13,14,15,16,25,29,30,31,32,38,39,40,41,42,43,45,46,47], suggesting an average calorie deficit of 479 kcal·day^−1^. It is important to note, however, the ranges reported for both energy expenditure (1744–3131 kcal·day^−1^) [11,12,14,30,31,32,38,41,43,46,47] and energy intake (1426–3122 kcal·day^−1^) [11,12,13,14,15,16,25,29,30,31,32,38,39,40,41,42,43,45,46,47] were considerably large and values are likely impacted by a combination of error in the methods used to predict/estimate both energy expenditure and energy intake, as well as the potential of underreporting by athletes [54,55].

Despite this, two studies [25,29] identified dietary intake in female soccer players that fell below the low energy availability cut-off of <30 kcal·kg FFM^−1^·day^−1^ [18,19,53] and from the two that provided comparisons of energy intake between training, competition, and recovery days [16,29], and no differences were observed despite increased energy demands. This provides more objective evidence to highlight that energy intake may be insufficient to meet needs and female field-based athletes may be at risk of the negative consequences associated with energy deficiency. Chronic energy deficiency can lead to insufficient glycogen stores and loss of fat-free mass, which can compromise performance through premature reductions in physical capacity and decreases in muscular strength and power [56,57]. From a health perspective, a long-term negative energy balance is likely to result in adaptations to reduce energy expenditure, prevent weight loss, and promote survival [55]. Unfortunately, such adaptations commonly result in impaired menstrual function and suboptimal bone health in female athletes and an increased tendency for injury and illness [17,18,19,21,27]. Increases in injury and illness rates can in turn have a further negative impact on sporting performance and training adaptation due to the prevention of consistent and high-quality training [58,59]. Risk of LEA has previously been associated with missing >22 days of training during the previous year due to illness and stress fractures [23].

The reason behind the observed lower energy intake in female field-based team sport athletes relative to their high energy demands remains unclear. However, it is likely to be multifaceted in nature. It has been suggested that athletes may inadvertently experience periods of low energy availability and compromised energy intake when the intensity and volume of training are high [55,60]. Under such conditions, athletes may simply be unaware of the energy cost of exercise [55], have a reduced number of eating occasions due to a demanding training schedule [61], and/or suffer from appetite suppression in response to high-intensity exercise [62,63]. Poor nutrition knowledge has previously been observed in female field-based team sport athletes [64,65] and this may also contribute to the lower energy intakes observed.

### 4.2. Carbohydrate Intake of Field-Based Team Sport Athletes

The average carbohydrate intake reported of 4.3 ± 1.2 g·kg·day^−1^ [11,12,13,14,15,16,25,29,30,31,32,38,39,40,41,42,43,45,46,47] falls below recommendations for a moderate exercise program (1h·day^−1^) of 5–7 g·kg·day^−1^. Despite an intake range between 2.7 and 8.3 g·kg·day^−1^, a majority of studies (*n* = 13) [11,12,13,14,15,16,29,30,31,38,39,40,43] reported carbohydrate intake below this recommendation. Akin to findings with regards to overall energy intake, a lack of carbohydrate periodization was also observed between training, recovery, and match-days [16,29]. These values suggest a systemic mismatch between the carbohydrate needs and intake in female field-based team sport athletes.

Given the intermittent high-intensity nature of field-based team sports, whereby players cover large total distances per game (6–10 km) [66,67,68], resulting in significant glycogen depletion [5], the maintenance of maximal performance in players is highly reliant on carbohydrate intake as a fuel source [4,69,70]. Across the course of a single soccer match glycogen depletion has been shown to occur and results in a reduction in distance covered, and speed of running during the second half of a match [71,72]. By extension, this could also impair players’ ability to perform repeated sprints, which is a performance-determining factor in intermittent team sports [73,74]. Soccer players that consumed a high-carbohydrate diet pre-match (65% TDEI) performed 30% more high-intensity runs than those who consumed a low-carbohydrate diet (30% TDEI) [75].

Deficiencies in carbohydrate intake and subsequent glycogen availability are thought to accentuate the stresses and negative consequences of energy deficiency and low energy availability [76]. Chronic low carbohydrate intake is related to a reduced capacity to use carbohydrate as a fuel, increased muscle breakdown, and impaired immune function [77]. Deficits as small as 10% in glycogen replenishment may lead to decreases in performance during subsequent training and/or competition and should be avoided [78]. Preservation of glycogen status has shown to diminish the hormonal impairments observed during periods of LEA [79], highlighting the importance of optimal carbohydrate intake in addition to meeting overall energy needs. Although this review highlights the poor carbohydrate intake in female field-based team sport athletes, the reason for this requires further investigation. A recent meta-analysis on male soccer players revealed a significant decrease in carbohydrate intake between the periods of 2000–2009 and 2010–2019 [80]. This may be a result of warnings about excessive carbohydrate intake leading to unwanted weight gain in the general population, and the more recent media-driven promotion of carbohydrate-restricted higher protein diets [76,80]. Interestingly, 47% of athletic and recreationally active females have reported previous adherence to a carbohydrate-restricted diet [23] and may explain the low carbohydrate intake observed in female field-based team sport athletes.

### 4.3. Protein Intake of Field-Based Team Sport Athletes

The average intake of protein reported (1.4 ± 0.3 g·kg·day^−1^) [11,12,13,14,15,16,25,29,30,31,32,38,39,40,41,42,43,45,46,47] falls within the protein recommendations (1.2–2.0 g·kg·day^−1^) [9] and is therefore likely to support recovery and maintenance/increase of muscle mass in female field-based team sport athletes [9,81]. It is important to note, however, that the co-ingestion of protein with carbohydrate has been shown to accelerate protein synthesis [82], therefore adequate protein intake alongside insufficient carbohydrate availability may diminish such a response. The minority of studies that reported protein intake below dietary recommendations provided values from off-season periods [25,31,39,45], which would be important to address if increases in muscle mass were a key goal during this timeframe. However, it is possible that protein intake may have been reduced purposefully in response to a decrease in training volume. Unfortunately, such information was unavailable, and studies that reported protein intake between training, recovery, and competition days [16,29] reported no significant difference. In respect of the range of protein intake recorded (0.9–2.0 g·kg·day^−1^) [11,12,13,14,15,16,25,29,30,31,32,38,39,40,41,42,43,45,46,47], those consuming intakes at the higher end of the scale may compromise their overall consumption of carbohydrate [83], and because of this, greater consumption of protein may indicate an increased risk of low energy availability [84].

### 4.4. Fat Intake of Field-Based Team Sport Athletes

Average intake of fat ranged from 21–37.5% of TDEI [11,12,13,14,15,16,25,29,30,31,32,38,39,40,41,42,43,45,46,47], with a minority of studies [12,30,31,40,43] reporting intakes that exceeded the recommended range of 20–35% [9]. In such scenarios, excessive fat consumption may compromise the overall consumption of carbohydrate (similarly to protein intake) as previously displayed within male soccer players [83]. From the limited studies that compared fat intake between training, recovery, and competition days [16,29], an increase in relative fat intake on a heavy training day in comparison to a light training day was observed with no significant changes in carbohydrate intake [29]. Dietary patterns that are high in fat but low in carbohydrate have been repeatedly shown to compromise high-intensity exercise performance [85] and would likely have a particularly detrimental impact on the performance of a field-based team sport athlete given their heavy reliance on carbohydrate as a fuel source [4,6].

### 4.5. Micronutrient Intake of Field-Based Team Sport Athletes

A majority of studies [11,13,15,16,42,45,46,47] reported intakes of calcium that meet recommendations of 700 mg·day^−1^ for females aged 19–50 years [33], whereas few [13,42,45,46] recorded intakes of iron that meet recommendations of 14.8 mg·day^−1^ for females aged 11–50 years [33]. The risk of iron deficiency in female athletes has been highlighted previously [26,27], and a combination of dietary inadequacy, declines in nutritional status due to heavy physical activity, and blood losses during menstruation periods are thought to contribute towards this [86,87]. The prevalence of iron deficiency among women competing in a variety of sports has previously been reported in the ranges of 25–35% [88], and 59% of the Swedish female national soccer team were found to be iron deficient before the 2003 FIFA women’s world cup [89]. If such iron deficiencies lead to iron deficiency anemia and the related decreases in circulating hemoglobin concentration, this would likely compromise exercise performance through a decrease in aerobic capacity [90]. Chronic iron deficiency anemia may also comprise an athlete’s general health due to fatigue, cognitive impairment, and suppressed immune system function [91]. Studies reported dietary intake of vitamin D that met recommendations of 10 µg·day^−1^ (400 IU·day^−1^) [34]. Despite most meeting the recommendation, 33–42% still appear to be deficient, highlighting dietary intake of vitamin D as a crude measure of vitamin D status [92]. Nonetheless, the low dietary intake of vitamin D, as observed in female field-based team sport athletes, may still be indicative of a requirement for vitamin D status to be assessed given the negative impact deficiency can have on bone density and stress fracture risk [26]. Micronutrient deficiencies have been reported to be accentuated by excessive exercise, restricted eating practices, and conditions of low energy availability [22,93,94], which, in conjunction with the observations presented, further highlights the requirement for micronutrient status to be regularly monitored among female field-based team sport athletes.

### 4.6. Sub-Group Comparisons

National/professional-level female field-based team sport athletes displayed similar dietary intake in comparison to their varsity/collegiate-level counterparts and therefore little difference was observed based on performance level. The average calorie intake of youth athletes (2304 ± 470 kcal·day^−1^) was higher than the average for adults (2018 ± 257 kcal·day^−1^), which could lead to greater energy deficits in adult athletes given their proportionally greater size and subsequent higher energy expenditure. The discrepancy in calorie intake between groups seemed to be primarily the result of greater carbohydrate intake in youth athletes (5.4 ± 1.7 g·kg·day^−1^) when compared to adults (4.1 ± 1.0 g·kg·day^−1^). This has been previously observed in male soccer players, whereby youth/junior players have displayed a greater % TDEI from carbohydrate [95] and higher carbohydrate intake overall [80], in comparison to adult/senior players. The reason for this remains unclear; however, the mean age of youth athletes included in this review (15.6 ± 2.0 years) may bring into question whether or not higher carbohydrate intakes were fulfilled on a self-determined and autonomous basis or instead were simply a consequence of support teams’/parental control over dietary intake. It is also important to acknowledge that the dietary intake data of adolescents can be particularly prone to misreporting [96]. Findings in reference to dietary intake observed across multiple time-points were equivocal, with three studies [31,32,39] reporting a lack of significant differences between phases in hockey and lacrosse players, while two studies [25,45] reported greater energy, carbohydrate, and protein intake during pre-season in comparison to in-season [25] and post-season [25,45] in soccer players. Unfortunately, all of the longitudinal investigations included [25,31,32,39,45] were limited to varsity/collegiate-level athletes and between-day observations for each phase were not reported. It would therefore be ambiguous to assume that the higher dietary intakes observed during pre-season periods [25,45] were meaningful attempts at nutritional periodization.

### 4.7. Limitations

The heterogeneity of studies included, and the limited reporting of health and performance outcomes prevented a meta-analysis from being performed and limited this review to a narrative synthesis. A large majority of field-based team sport athletes included within this review were female soccer players and findings may be less relevant to field-based sports where only small sample sizes were captured. Many of the studies included relied on prospective food records, which are known to influence usual intake, and are prone to both under and over-reporting [61,97]. A previous meta-analysis reported a mean bias of 19% underreporting (600 kcal·day^−1^) when comparing self-reported methods to doubly labelled water techniques [54]. Many of the studies included within this review highlighted the potential for underreporting within their data and utilized a broad range of methods to measure both energy intake and energy expenditure, limiting the extent of comparison that can be made. This, however, speaks to a larger issue that standardized techniques to measure energy intake, energy expenditure, and energy availability in free-living athletes are yet to be determined, and all are subject to a degree of error [55]. However, underreporting and error alone cannot refute the substantial body of evidence presented by this review that indicates that female field-based team sport athletes’ diets are deficient in both overall energy and carbohydrate intake. Future research would benefit from adopting standardized techniques to measure dietary intake with longitudinal observations of professional/elite female field-based team sport athletes and reporting of between-day differences of both energy intake and expenditure. Future investigations should also aim to capture factors that might influence dietary intake, such as nutrition knowledge [98,99], and assess both the risk factors and negative health outcomes of low energy availability that may also be present, using validated tools for both [100,101,102].

## 5. Conclusions

This review identified that in comparison to dietary recommendations for health and sports performance, female field-based team sport athletes present diets that are insufficient in overall energy, carbohydrate, and iron intake. When interpreted in the context of the high energetic demands of field-based sports, female athletes may be viewed as a particularly high-risk group for low energy availability and its associated health implications. Future research is required to establish the reason behind current dietary practices observed and to explore the potential negative consequences athletes might experience as a result. Based on this review’s findings, interventions to promote greater adherence to dietary recommendations in female field-based team sport athletes are recommended to prevent the potential negative health consequences and performance impairments related to inadequate dietary intake. These interventions should measure the influence of improvements in dietary intake on a broad array of health and performance outcomes as this review highlights that such information is currently lacking.

## Figures and Tables

**Table 1:** Participants, intervention, comparison, outcome, and study (PICOS) criteria.

Parameter	Description
Population	Female field-based team sport athletes
Intervention/exposure	Baseline/habitual dietary intake
Comparison	Dietary intake in comparison to consensus recommendations
Outcomes	Meeting/not meeting recommendations
Study design	Cross-sectional, longitudinal, and randomized controlled trials

**Table 2:** Search terms.

Concept	Keywords
Dietary Intake	“nutrient requirement*” OR “dietary intake*” OR “daily food*” OR “food intake*” OR “dietary assessment” OR “dietary requirement*” OR “sports nutrition” OR “sport nutrition” OR “food diary” OR “food frequency” OR “macronutrient” OR “nutrient needs” OR “dietary needs” OR “nutrient intake” OR “RDA”
Field-Based Team Sport	Rugby OR Soccer OR Football OR “Australian Rules” OR Hurling OR Camogie OR Hockey OR “Field Hockey” OR Lacrosse OR “Gaelic*” OR “GAA” OR “field sport*” OR “field-sport*” OR “field-based sport*” OR “field based sport*” OR “field-sport athlete*” OR “field sport athlete*” OR “team-sport” OR “team sport*” OR “team-based sport*” OR “team based sport*” OR “team-sport players” OR “team sport players” OR “team-sport athlete*” OR “team sport athlete*” OR “invasion-team sport*” OR “invasion team sport*” OR “invasion sport*”

**Table 3:** Eligibility criteria.

Inclusion	Exclusion
Original research (cross-sectional, observational, randomized controlled trials)	Reviews and other secondary research
English language studies	Non-English language studies
Female field-based team sport athletes	Male and/or non-field-based team sport athletes
Competitive athletes (youth/adult, professional/amateur)	Recreational only (non-competitive)
Reporting of baseline/habitual dietary intake	Reporting of dietary intake only after an intervention
Quantitative assessment methods used (24-h recall, weighed food records)	Qualitative assessment only (interviews, focus groups)

**Table 4:** Study characteristics.

Ref	Participant Characteristics							Dietary Intake Data									
Author, Year	Sport	Level	Phase	N	Mass (kg)	Height (cm)	EE (kcal·day^−1^)	EI (kcal·day^−1^)	CHO (g)	CHO (g·kg·day^−1^)	Protein (g)	PRO (g·kg·day^−1^)	Fat (g)	Fat (%)	Calcium (mg·day^−1^)	Iron (mg·day^−1^)	Vit D (µg·day^−1^)
Braun et al. (2018) [47]	S	National-Youth	IS	56	56.8 ± 6.1	166 ± 5	2403 ± 195	2262 ± 368	303 ± 62	5.4 ± 1.1 *	77 ± 11	1.4 ± 0.3 **	78 ± 19	30.7 ± 5.0 †	1107 + 382	14 ± 2.7	1.3 ± 0.9
Clark et al. (2003) [45]	S	NCAA 1	PRS	13	62.0 ± 4.8	166 ± 5	N/A	2290 ± 310	320 ± 70	5.2 ± 1.1 *	87 ± 19	1.4 ± 0.3 **	75 ± 13	29.0 ± 5.7 †	931 ± 223	17.3 ± 4.7	2.4 ± 1.7
			POS	13	61.6 ± 4.7	166 ± 5	N/A	1865 ± 530	263 ± 71	4.3 ± 1.2	59 ± 17	1.0 ± 0.3	66 ± 29	31.0 ± 6.6 †	695 ± 289	12.2 ± 5.2	2.5 ± 2.6
Condo et al. (2019) [15]	ARF	PL	PRS	29	64.5 ± 8.0	168 ± 8	N/A	1870 ± 577	192 ± 52	3 ± 0.8	98 ± 32	1.5 ± 0.5 **	72 ± 33	33.2 ± 6.5 †	925 ± 545	12.2 ± 3.2	N/A
Dobrowolski et al. (2019) [14]	S	PL	IS	41	62.5 ± 9.8	168 ± 5	2811 ± 493	1476 ± 434	199 ± 21	3.3 ± 1.2	72 ± 24	1.2 ± 0.4 **	47 ± 21	28.8 †	646 ± 290	8.8	1.69
dos Santos et al. (2016) [41]	S	PL	PRS	21	56.9 ± 6.3	162	2701 ± 214	2306 ± 405	311	5.5 ± 0.9 *	114	2 ± 0.5 **	67	26.3 ± 5.6 †	N/A	N/A	N/A
Gibson et al. (2011) [46]	S	Elite-Youth	IS	33	60.9 ± 8.2	164 ± 6	2546 ± 190	2079 ± 460	294 ± 85	5.0 ± 1.6 *	82 ± 19	1.4 ± 0.3 **	69 ± 20	29.9 ± 5.8 †	931 ± 351	16.2 ± 5.9	4.1 ± 2.4
Gravina et al. (2012) [40]	S	PL	IS	28	61 ± 8.4	N/A	N/A	2271 ± 578	252	4.1	85	1.4 **	93	37 ± 7	N/A	N/A	3.3 ± 2.0
Hosseinzadeh et al. (2017) [42]	S	Elite-Youth	IS	8	53.3 ± 11.3	160 ± 5	N/A	3122 ± 746	445 ± 93	8.3 *	86 ± 17	1.6 **	110 ± 32	31.7 †	1197 ± 451	29.7 ± 11.4	5.4 ± 1.8
Jagim et al. (2019) [12]	LX	NCCA 2	IS	20	69.9 ± 10.7	170 ± 6	2582 ± 303	2161 ± 392	236 ± 74	3.5 ± 1.2	79 ± 20	1.2 ± 0.4 **	88 ± 23	36.6	N/A	N/A	N/A
Jenner et al. (2019) [16]	ARF	PL	PRS	23	67 ± 8	169 ± 7	N/A	1884 ± 457	178 ± 44	2.7 ± 0.7	107 ± 33	1.6 ± 0.5 **	73 ± 23	34 ± 6.0 †	852 ± 288	12.1 ± 3.5	N/A
Kumahara et al. (2020) [32]	LX	C	OS	17	52.5 ± 5.6	159 ± 6	1744 ± 138	1847 ± 398	257	4.9 ± 0.9	58	1.1 ± 0.3	63	30.7 ± 3.9 †	436 ± 127	6.3 ± 2.1	4.6 ± 2.3
			PRS	17	53.0 ± 5.3	159 ± 6	2168 ± 248	2020 ± 426	279	5.2 ± 1.1 *	64	1.2 ± 0.3 **	72	32.2 ± 3.9 †	500 ± 189	6.8 ± 1.6	4.1 ± 1.8
Marsh et al. (2017) [43]	TR	I	CP	16	60 ± 6.5	163 ± 6.0	2616	2394	264	4.4	120	2 **	98	36.7	N/A	N/A	N/A
Martin et al. (2006) [11]	S	I	IS	16	61.5 ± 5.3	167 ± 8.0	2154 ± 596	1904 ± 366	256	4.1 ± 1.0	74	1.2 ± 0.3 **	61	29.0 ± 6.6 †	840 ± 335	12.1 ± 6.0	2.1 ± 0.8
Moss et al. (2020) [29]	S	PL	IS	13	63.7 ± 7.0	169 ± 8.0	N/A	2124 ± 444	211 ± 46	3.3 ± 0.6	117 ± 30	1.8 ± 0.4 **	89 ± 30	21 ± 4 †	N/A	N/A	N/A
Mullinix et al. (2002) [13]	S	I	NS	11	59.7 ± 7.1	162 ± 13	N/A	2015 ± 19	282 ± 118	4.7	79 ± 33	1.3 **	67 ± 28	29.9 †	887 ± 510	16 ± 7.8	2.6 ± 2.4
Nutter (1991) [39]	FH	C	IS	9	63.5 ± 6.2	163 ± 6.0	N/A	1513 ± 406	204	3.2	60	1.0 ± 0.3	45	27 ± 9 †	N/A	N/A	N/A
			POS	9	63.8 ± 5.5	163 ± 6.0	N/A	1426 ± 394	193	3.0	57	0.9 ± 0.3	48	30 ± 7 †	N/A	N/A	N/A
Reed et al. (2014) [25]	S	NCAA 1	PRS	19	60.8	166	N/A	2390	392	7.0 *	104	2.0 **	93	29 †	N/A	N/A	N/A
			IS	15	62.4	166	N/A	2029	292	5.0 *	91	2.0 **	78	31 †	N/A	N/A	N/A
			POS	17	N/A	N/A	N/A	2117	272	5.0 *	84	1.0	77	31 †	N/A	N/A	N/A
Wardenaar et al. (2017) [38]	S	National-Youth	IS	16	58.4 ± 6.1	168 ± 7	2557	1965	256 ± 31	4.3 ± 0.5	76 ± 4	1.3 ± 0.1 **	66 ± 13	30 †	N/A	N/A	N/A
	RS	National	IS	29	66.5 ± 6.1	169 ± 5	3131	2056	244 ± 44	3.5 ± 0.6	93	1.4 ± 0.1 **	71 ± 10	31.6 †	N/A	N/A	N/A
	FH	National-Youth	IS	11	61.7 ± 4.7	169 ± 4	2796	2091	259 ± 37	4.2 ± 0.5	90 ± 10	1.4 ± 0.1 **	70 ± 20	29.6 †	N/A	N/A	N/A
Yli-Piipari (2019) [30]	S	NCAA 1	IS	13	59.9 ± 4.9	167 ± 5	2486 ± 208	1895 ± 428	252	4.2 ± 2.3	93	1.6 ± 1.1 **	79	37.5	N/A	N/A	N/A
Zabriskie et al. (2019) [31]	LX	NCAA 2	OS	20	68.8 ± 8.9	168 ± 7	2603 ± 378	2242 ± 462	262 ± 61	3.9 ± 1.1	80 ± 19	1.2 ± 0.3 **	78 ± 20	31.3 †	N/A	N/A	N/A
			OS	20	69.6 ± 9.5	168 ± 7	2579 ± 376	2015 ± 451	231 ± 59	3.4 ± 0.9	72 ± 20	1.1 ± 0.3	70 ± 25	31.3 †	N/A	N/A	N/A
			PRS	20	69.6 ± 10.0	168 ± 7	2798 ± 391	2079 ± 435	247 ± 74	3.6 ± 1.2	82 ± 22	1.2 ± 0.4 **	74 ± 23	32 †	N/A	N/A	N/A
			IS	20	69.3 ± 10.0	168 ± 7	2513 ± 248	2124 ± 505	248 ± 66	3.6 ± 0.9	84 ± 16	1.2 ± 0.3 **	81 ± 26	32.9 †	N/A	N/A	N/A
			IS	20	68.9 ± 10.1	168 ± 7	2582 ± 303	2161 ± 392	236 ± 74	3.5 ± 1.2	79 ± 20	1.2 ± 0.4 **	88 ± 23	36.7	N/A	N/A	N/A

Note: Data are mean and SD (where available), S Soccer, ARF Australian Rules Football, PL Professional League, LX Lacrosse, C Collegiate, TR Touch Rugby, I International, NS Not Specified, FH Field Hockey, RS Rugby Sevens, IS In Season, OS Off Season, PRS Pre-Season, POS Post-Season, CP Competition, EE Energy Expenditure, EI Energy Intake, CHO Carbohydrate, PRO Protein, N/A Non-applicable/not reported,* meets carbohydrate recommendations for a moderate exercise program (1 h·day^−1^) of 5–7 g·kg·day^−1^ [10,11], ** meets protein recommendations of 1.2–2.0 g·kg·day^−1^ [11], † meets fat recommendations of 20–35% total daily energy intake [11].

**Table 5:** Dietary intake comparison between sports.

Sport	N	EI (kcal·day^−1^)	CHO (g·kg·day^−1^)	PRO (g·kg·day^−1^)	Fat (% EI)
Soccer	288	2132 ± 347	4.9 ± 1.3	1.5 ± 0.3 *	30.2 ± 3.7 **
Lacrosse	57	2081 ± 122	4.0 ± 0.7	1.2 ± 0.1 *	33.0 ± 2.4 **
Australian Rules	52	1877 ± 10	2.9 ± 0.2	1.6 ± 0.1 *	33.6 ± 0.6 **
Field Hockey	20	1677 ± 361	3.5	1.1 ± 0.3	28.9 ± 1.6 **
Rugby Sevens	29	2056	3.5	1.4 *	31.6 **
Touch Rugby	16	2394	4.4	2.0 *	36.7

Note: Data are mean and SD (where available), Soccer [11,13,14,25,29,30,38,40,41,42,45,46,47], Lacrosse [12,31,32], Australian Rules [15,16], Field Hockey [38,39], Rugby Sevens [38], Touch Rugby [43], EI Energy Intake, CHO Carbohydrate, PRO Protein, N/A Non-applicable/not reported, * meets protein recommendations of 1.2–2.0 g·kg·day^−1^ [11], ** meets fat recommendations of 20–35% total daily energy intake [11].
